# Impact Toughness of Hybrid Carbon Fiber-PLA/ABS Laminar Composite Produced through Fused Filament Fabrication

**DOI:** 10.3390/polym13183057

**Published:** 2021-09-10

**Authors:** Hafiz Ahmed, Ghulam Hussain, Sohail Gohar, Aaqib Ali, Mohammed Alkahtani

**Affiliations:** 1 Faculty of Mechanical Engineering, Ghulam Ishaq Khan Institute of Engineering Sciences and Technology, Topi 23460, Pakistan; ahmedgik16@gmail.com (H.A.); sohailgohar989@gmail.com (S.G.); 2 Department of Mechanical Engineering Technology, Punjab Tianjin University of Technology, Lahore 54770, Pakistan; 3 Department of Mechanical Engineering, Michigan State University, East Lansing, MI 48824, USA; aliaaqib@msu.edu; 4 Industrial Engineering Department, College of Engineering, King Saud University, Riyadh 11421, Saudi Arabia

**Keywords:** hybrid laminar composites, Fused Filament Fabrication, toughness, 3D printing parameters, optimization

## Abstract

Nowadays, the components of carbon fiber-reinforced polymer composites (an important material) are directly produced with 3D printing technology, especially Fused Filament Fabrication (FFF). However, such components suffer from poor toughness. The main aim of this research is to overcome this drawback by introducing an idea of laying down a high toughness material on the 3D-printed carbon fiber-reinforced polymer composite sheet, thereby making a hybrid composite of laminar structure. To ascertain this idea, in the present study, a carbon-reinforced Polylactic Acid (C-PLA) composite sheet was initially 3D printed through FFF technology, which was then laid upon with the Acrylonitrile Butadiene Styrene (ABS), named as C-PLA/ABS hybrid laminar composite, in an attempt to increase its impact toughness. The hybrid composite was fabricated by varying different 3D printing parameters and was then subjected to impact testing. The results revealed that toughness increased by employing higher layer thickness and clad ratio, while it decreased by increasing the fill density, but remained unaffected due to any change in the raster angle. The highest impact toughness (23,465.6 kJ/m^2^) was achieved when fabrication was performed employing layer thickness of 0.5 mm, clad ratio of 1, fill density of 40%. As a result of laying up ABS sheet on C-PLA sheet, the toughness of resulting structure increased greatly (280 to 365%) as compared to the equivalent C-PLA structure, as expected. Two different types of distinct failures were observed during impact testing. In type A, both laminates fractured simultaneously without any delamination as a hammer hit the sample. In type B, the failure initiated with fracturing of C-PLA sheet followed by interfacial delamination at the boundary walls. The SEM analysis of fractured surfaces revealed two types of pores in the C-PLA lamina, while only one type in the ABS lamina. Further, there was no interlayer cracking in the C-PLA lamina contrary to the ABS lamina, thereby indicating greater interlayer adhesion in the C-PLA lamina.

## 1. Introduction

Laminar composites are made up of a series of lamina. By the choice of laminar architecture (like volume percent of the constituent materials and layer thickness) and constituent materials, laminar composites can be engineered to prepare a structure with required properties [1]. Upon loading, the delamination of different layers is one of the main failure modes of laminar composites. Various ply-stitching techniques are employed to avoid delamination. Laminar composites are widely used in aerospace, automobile, and defense equipment due to their high stiffness, strength, low density, and resistance to corrosion [2].

Hand lay-up, filament winding, and compression molding are the common conventional methods to fabricate laminar composites [3,4]. Blades of wind turbines, pressure vessels, gas cylinders, tanks, automobile panels, and bumpers are prepared using these techniques. In hand lay-up, the resins are infused manually by the help of rollers and brushes into fibers, which are in the form of stitched, bonded, knitted, or woven fabrics. However, uniformity and consistency cannot be achieved in this method. Filament winding is used for manufacturing hollow, oval, and circular sectioned products like pressure vessels, casings, gas cylinders, etc. [5,6]. Only convex shape components can be manufactured using this technique. Whereas in compression molding, heat and pressure are applied for specific time to manufacture different laminar composites [7]. The use of dies and molds in compression molding increases the lead time and production cost. This technique is only feasible for high volume production and needs manpower for finishing of the product.

On the other hand, Fused Filament Fabrication (FFF), an innovative process, does not suffer from the aforementioned issues because it offers flexible and dye-free fabrication [8,9]. It is one of the 3D printing techniques in which extruder gets the filament from the spool and material is melted in a heated nozzle and deposited in the form of layers on top of a heated platform [10,11,12,13]. The main advantage of FFF technology is to produce quick prototyping polymer samples [14,15]. The use of FFF technology has been increased in many applications due to its easy use, simplicity, low lead time, and cost effectiveness [14,16,17]. FFF also has many applications in the field of aviation, design verification, medicine, tissue engineering, and prosthetics [18,19,20]. In fact, studies have shown some advantages of FFF over conventional polymer processing techniques for enhancing material performance, e.g., impact strength of 3D printed PLA (printed using layer thickness of 0.2 mm, and plate temperature of 160 °C) is better than that of the injection molded PLA [21].

Attempts have been made to enhance the mechanical properties of 3D-printed components with the addition of particles and fibers. This technique has been employed in the polymer industries to increase the structural strength of the 3D-printed components [22]. Zhong et al. [23] reinforced ABS filament with short glass fibers and on 3D printing, they observed an increase in the strength in comparison to unreinforced ABS filament. Hassan and Jwu [24] prepared filaments by blending polycarbonate (PC) with ABS. It was depicted that impact toughness increased by increasing the amount of PC. Tekinalp et al. [25] prepared composites using carbon fiber-reinforced ABS as 3D printing material and concluded that carbon fiber reinforced ABS offered better mechanical properties than unreinforced ABS. They also found that impact strength increased by decreasing the carbon fiber content. Kannan et al. [26] fabricated Ni-coated ABS plastics and it was determined that impact energy of the Ni-coated ABS was improved.

Carbon fiber-reinforced polymer composites are gaining popularity owing to their extensive high-end applications. Nowadays, it has become a common practice to build carbon fiber-based polymer components using the FFF technique. Although such composite components exhibit high strength, these suffer from poor toughness. Various studies have been carried out to investigate impact toughness of FFF-printed materials. Vidakis et al. [27] performed experiments on ABS and ABS-plus samples using notched and unnotched specimens for standard Charpy’s impact test. They found that impact strength of 3D-printed specimens was lower than the parent bulk materials. Morales et al. [28] analyzed the quasi-static and dynamic crush behavior of 3D-printed thin-walled hollow profiles made using polyamide matrix reinforced with continuous carbon (cCF/PA) and glass fibers (cGF/PA), and found that strain-hardening effect enhanced impact resistance of cGF/PA material, but not of cCF/PA. Fekete et al. [29] proposed to blend PLA with rubber (up to 20 wt%) to enhance impact toughness after printing.

The FFF-built carbon fiber-based polymeric composites reported so far have been mainly fabricated as monolithic/single sheets [21,30]. As carbon fiber is a brittle material, one of the shortcomings of these monolithic composite sheets is thus their low impact toughness. The laminar structures, on the other hand, are believed to offer several benefits including high fracture toughness and outstanding specific strength [31]: properties highly desired in mechanical structures [32,33]. The low toughness of the mentioned material, therefore, can be raised by coupling it with the laminate of a tougher material. 3D-printed composites with laminar structure, produced especially through FFF technology, are scarcely reported in the literature. Liu et al. [34] employed 3D printing (bio-plotter) to produce hybrid bi-layer (polycaprolactone/Gel/nano-hydroxyapatite) scaffold for guided bone regeneration. Khan et al. [33] studied the interfacial bonding between two laminates in different delamination modes. They made the laminates with Acrylonitrile Butadiene Styrene (ABS) and Carbon-fiber reinforced Polylactic Acid (C-PLA) by varying the printing parameters. The results showed that the printing speed and nozzle temperature are the important parameters affecting the interfacial bonding. This is reasoned to the fact that these parameters influence fusion of the layers, the main mechanism that controls interfacial bonding and relies on heat available during bonding, which further depends on the printing parameters.

From the above literature analysis, it is concluded that impact toughness of the FFF-built laminar composites is not reported in the literature. Further, the idea to enhance the toughness of carbon fiber-based polymer composites by overlaying it with a tougher polymer is yet unexplored. The present study is aimed at developing a laminar composite (composed of two laminates) and characterizing its impact toughness in order to examine usefulness of the proposed idea. The laminates are fabricated in two filament materials, namely, Carbon-fiber reinforced Polylactic Acid (C-PLA) and Acrylonitrile Butadiene Styrene (ABS), thus resulting in a laminar hybrid composite. The reason of choosing C-PLA is that it is a carbon-filled composite filament with a high strength and stiffness available commercially. The ABS was selected as it is a polymer alloy with a reasonable mix of various mechanical properties and high toughness, thereby supplementing low toughness of C-PLA lamina. The hybrid composite is made by varying boundary wall configuration and printing parameters, namely, layer thickness (LT), raster angle (RA), fill density (FD), and clad ratio (CR). The toughness of the printed composite samples is determined by performing an impact test. The results confirm that superimposing ABS on C-PLA lamina can substantially raise the toughness of C-PLA sheet. Moreover, an optimum set of parameters yielding high toughness is also proposed.

## 2. Preliminary Experiments and Methodology

### 2.1. Materials

Two types of filament materials were employed, namely, ABS and C-PLA. True white ABS filament is an alloy of three polymers, namely, acrylonitrile, butadiene, and styrene. C-PLA is a composite filament prepared by mixing 4043D PLA resin with chopped short carbon fibers (15% wt) (Make: ProtoPlant, USA). Both of these filaments had a diameter of 1.75 mm (+0.5 mm). Their properties are listed in Table 1 [35,36]. These materials were purchased from the suppliers. ABS filament was supplied by Xplorer 3D (Dubai, UAE), and C-PLA was supplied by ProtoPlant, USA.

The 3D printing temperatures and bed temperatures for ABS are 220–230 °C and 80–90 °C, respectively. For C-PLA, these temperatures are 190–210 °C and 50 °C, respectively [37]. C-PLA is a long-lasting filament with excellent layer adhesion ability and structural strength. Carbon fiber in the filament provides excellent structural support due to its high rigidity. On the other hand, ABS is very durable, less brittle, and more flexible than PLA, and has the ability to survive high temperatures. C-PLA and ABS are easily 3D printable materials with good mechanical properties. Some important properties of ABS and C-PLA are listed in Table 1 [36].

### 2.2. Test Geometry and Its Fabrication

**Figure 1:**
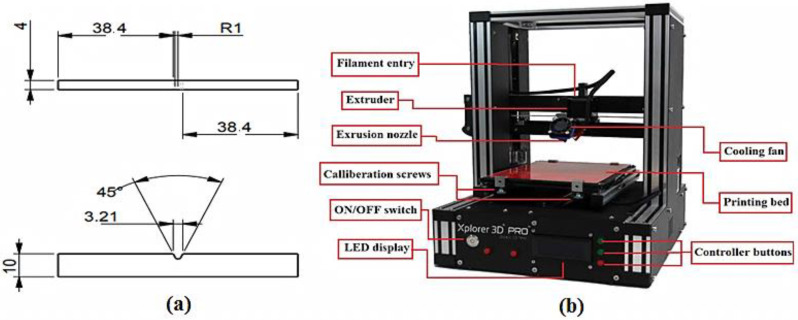
(**a**) Standard sample for impact testing (**b**) Xplorer 3D Pro.

**Figure 2:**
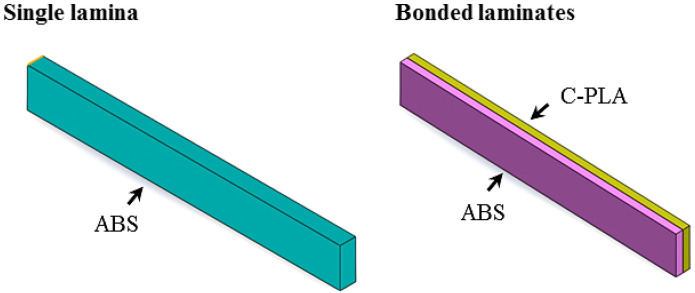
Schematic showing single and two bonded laminates: both types have same size. Thickness of each lamina in bonded laminates varies depending on the clade ratio as given in Section 2.4.

The impact test geometry, according to ISO 179-1 standard, employed in this study is shown in Figure 1a. The “Xplorer 3D Pro (Dubai, UAE)” printer (Figure 1b) was used to fabricate these specimens. Two types of impact specimens were made: as a single (or monolithic) lamina and as two bonded laminates (Figure 2), both having the same size, including thickness of 4 mm. The maximum 3D printing volume for this printer was 200 mm × 200 mm × 200 mm. The specimens were printed by extruding the filament polymer through heated nozzle and laying down the molten polymer onto the heated bed in series of layers (see a representative sample in Figure 2). For making a specimen in a single sheet, only one type of filament (say ABS or C-PLA) was used to print the required thickness. To manufacture specimen as two bonded sheets (i.e., laminar composite), initially the C-PLA layers were deposited using the C-PLA filament (a composite filament). After laying down its required number of layers, ABS layers were deposited over a printed sheet of C-PLA without any interruption, making the total thickness of 4 mm. The thickness of each of the C-PLA and ABS laminates was maintained according to the clad ratio discussed in Section 2.4. While printing of C-PLA lamina was nearing completion, the supply of C-PLA filament was cut to replace it with the ABS filament in order to lay down the ABS lamina onto the previously printed C-PLA lamina. Thus, this way of printing resulted into the C-PLA/ABS hybrid laminar composite. When a layer is deposited, it cools down and solidifies quickly. During the 3D printing process, the material in each layer diffuses with the previous layer or adjacent material due to thermal bonding. The local welding of the printed layers and ability of fusion bonding to manufacture components with good control of their properties is one of the prominent characteristics of the FFF [38].

### 2.3. Preliminary Experiments

**Figure 3:**
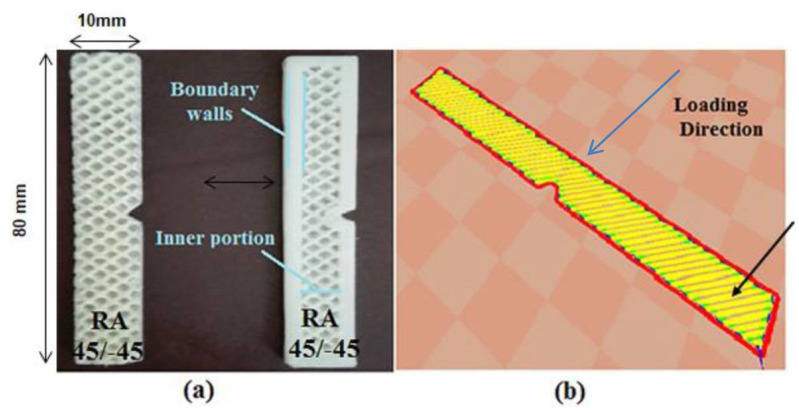
(**a**) Impact sample without and with boundary wall (**b**) Loading direction perpendicular to boundary wall.

The impact samples can be 3D printed either with boundary wall or without boundary wall. Therefore, some preliminary tests were performed to explore the effect of boundary wall. Individual specimens of ABS, C-PLA and ABS/C-PLA hybrid composite were made using the FD of 40% and 100%, feed of 20mm/s and LT of 0.5 mm. Figure 3a shows the sample with and without boundary walls. The direction of load is perpendicular to the boundary wall of the samples (Figure 3b). The impact tests were carried out on Shimadzu Charpy impact testing machine (Shimadzu, Kyoto, Japan). Each test was performed twice and the mean value was used for analysis.

Impact energy can be found by following equations:

Impact Energy (E) = m × g × L × (cos α − cos β) (1)

Impact Force (F) = (h_1_ − h_2_) × g × m (2)

(3) $$\mathrm{Impact}\;\mathrm{toughness}=\frac{E}{A}$$

where, m = mass of hammer, g = gravitational acceleration, L = length of hammer, α = Angle of hammer’s initial position, β = Angle of hammer’s final position, h_1_ = Initial height of hammer, h_2_ = Final height of hammer after striking, *E* = Impact energy to fracture the specimen, and *A* = cross-sectional area.

**Figure 4:**
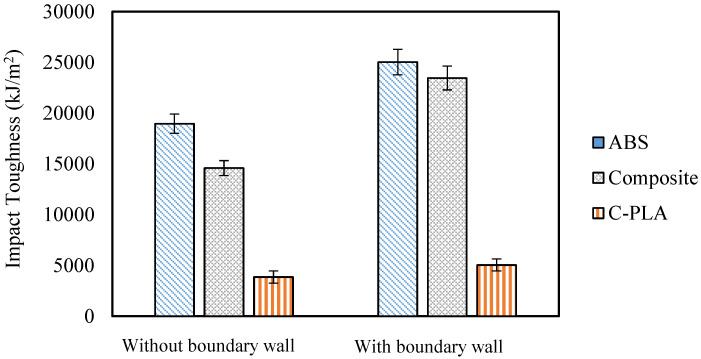
Boundary wall effect on impact toughness considering various materials: ABS, C-PLA, and hybrid laminar composite: each test was repeated twice.

Figure 4 depicts that the impact toughness of ABS lamina, C-PLA lamina and hybrid composite specimens made with boundary wall is higher in comparison to the corresponding specimens made without boundary wall. The improvement in the impact toughness due to boundary wall can range up to 60%, as presented in Table 2. This improvement occurs because the boundary wall bears the major portion of load, while the core experiences a lesser load. As the specimens with boundary wall showed better results, so samples for further investigation were 3D printed with boundary wall. 

### 2.4. Experimental Plan

**Figure 5:**
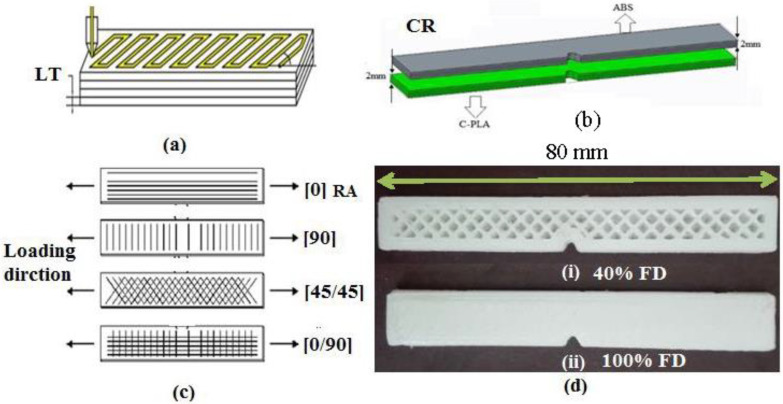
Four different 3D printing parameters: (**a**) layer thickness, (**b**) clad ratio, (**c**) raster angle, and (**d**) fill density.

To investigate the effect of 3D printing parameters on the impact toughness of the hybrid laminar composite, four different 3D printing parameters, i.e., LT, FD, RA, and CR were varied: see definitions in Figure 5a–d. The fixed 3D printing parameters were printing speed = 20 mm/s, shell thickness = 2 mm, and nozzle diameter = 1 mm. Lower printing speed increases inter-lamina bonding [33]; hence, it was kept fixed to a low value. Preliminary tests, discussed earlier, showed that shelled (with boundary) specimens offer more impact toughness than non-shelled does. Therefore, shell thickness was kept to a reasonable value of 2 mm. As regards to the nozzle diameter, it was kept fixed because more options were not available on the printer used herein in the study. The range of variable and fixed parameters was decided according to the results found in literature [39,40,41,42]. LT lower than 0.1 mm increases number of layers. Larger number of layers is not admirable in impact testing specimens, as excessive thermal cycles cause interlayer cracking. LT greater than 0.5 mm could not be achieved due to limitation of 3D printer. FD was set at 40 and 100%, because FD lesser than 40% would make unduly porous structures. Since the RA has been reported as an influential 3D printing parameter [36], two types of RA were selected, i.e., 0°/90° and 45°/−45°. In this research, a new 3D-printing parameter known as clad ratio (CR) is also investigated. The value of CR decides the fraction of two parent materials to form a laminar composite. CR is ratio of thickness of ABS to the thickness of C-PLA. If CR is 1, it means that laminar composite is made up of 50% ABS and 50% C-PLA. If CR = 0.5, it depicts that composite has 67% of C-PLA and 33% of ABS as it can be calculated by simultaneously solving the following equations:

CR (0.5, 1) = ABS/C-PLA(4)

ABS + C-PLA = 4 (Specimen Thickness)(5)

A comprehensive test plan was prepared using Design Expert (DX-10) software. Full factorial design was adopted for this experimentation as it explores many factors, setting each factor to only two levels. Full factorials design is useful for the estimation of individual and interactive effects. The mathematical form of design is shown below:

X^n^ = 2^4^ = 16 (6)

where

X = No. of levels
sn = No. of parameters

The response is dependent on various independent variable inputs. By using the minimum and maximum values of the 3D printing parameters, the test plan of sixteen test runs is shown in Table 3. Each test was performed twice and the mean value was used for results.

### 2.5. Fractured Surfaces Characterization

In order to analyze the behavior of fractured surfaces, PHILIPS XL-30 with tungsten filament SEM was employed. The surface of polymer sample should be conductive to perform SEM. For this purpose, sputtering was done on the samples. In sputtering, vacuum pressure of 10^−2^ was created to remove residual gases for reaction. Current and voltage of 10–15 mA and 1–1.4 KV were maintained, respectively. Conductive layer of copper was sprayed on the fractured surfaces of polymers.

## 3. Results and Discussion

Table 3 presents the impact toughness results, a response variable in the statistical analysis. The highest value of impact toughness for the C-PLA/ABS laminar composite was found to be 23,465.6 kJ/m^2^ for test No. 3 against the following 3D printing parameters: LT = 0.5mm, RA = 45°, CR = 1 and FD = 40%. While the lowest value of impact toughness was 7672.8 kJ/m^2^ for test No. 14 against the following 3D printing parameters: LT = 0.1 mm, RA = 0°, CR = 0.5, and FD = 100%.

The percentage improvement in impact toughness due to laying ABS on C-PLA sheet is presented in Table 4. The impact toughness of bi-sheets laminar composite (C-PLA/ABS) and equivalent monolithic C-PLA sheet was determined, employing the same printing and testing conditions. As can be noticed, an increase ranging from 280 to 365% is obtained by adopting the proposed idea.

Analysis of Variance (ANOVA) was performed on the results in order to identify the significant parameters affecting the impact toughness of the composite. A parameter with *p*-value ≤ 0.05 (95% confidence level) was regarded as significant. This can be noticed from Table 5 that LT with *p*-value < 0.0001 is the most significant 3D printing parameter.

### 3.1. Effects of 3D Printing Parameters on Impact Toughness

**Figure 6:**
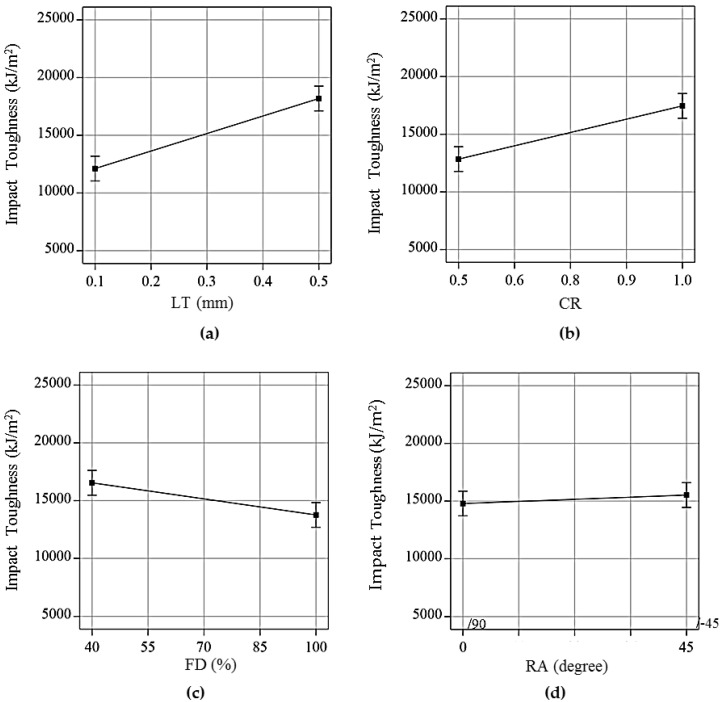
Effect of printing parameters on impact toughness of hybrid laminar composite: (**a**) layer thickness, (**b**) clad ratio, (**c**) fill density, and (**d**) raster angle.

Figure 6a shows that impact toughness of the hybrid composite increases as the LT increases. This is due to the fact that the material experiences lesser number of thermal cycles, which in turn minimizes the likelihood of interlayer cracking [43]. Moreover, at higher LT, each layer has better homogeneity within itself, which leads to uniform molecular bonding, therefore offering greater resistance to the penetration [41]. As a result, this enables the material to withstand greater impact energy leading to higher impact toughness. This finding is in agreement with Santhakumar et al. [44] and Shubham et al. [41], who 3D printed monolithic composites of Polycarbonate and ABS, respectively. 

Figure 6b depicts the effect of CR on the impact toughness. There was an increase in the impact toughness with the increase in the CR. In fact, an increase in the CR means a corresponding increase in the volume fraction of ABS material. Being ductile than the C-PLA, the ABS material has tendency to absorb more impact energy than C-PLA. As a result, the impact toughness of ABS/C-PLA composite increased as the CR increased. 

Contrary to the effects of LT and CR, Figure 6c illustrates that the FD has inverse relation with the impact toughness, i.e., impact toughness increased with the decrease in the FD. This may be reasoned to the fact that the porosity in the composite structure increases with the reduction in FD, which improves the ability of material to absorb impact energy at failure. This finding agrees with Joseph et al. [45] who studied the influence of varying FD on the impact toughness of monolithic composite sheets. Isfahani et al. [46] performed impact tests on hollow, solid, and hybrid polyester fiber composites. They concluded that any decrease in the FD creates hollow spaces, which reduces the density of fiber and improves the ability of energy absorption, consequently increasing the impact toughness. The present findings are in agreement with Isfahani et al. [46] and can be attributed to increased energy absorption ability due to increase in porosity in the structure. 

Figure 6d shows that the RA did not have any substantial effect on the impact toughness. On the other hand, it has been reported as an influential parameter for tensile strength of FFF structures [47,48]. In fact, the specimens in this specific case were fabricated with a boundary wall with a fixed raster angle of 0°. The raster angle was changed just in the inner portion of the specimen, as shown in Figure 6d. From the results, it seems that change in the raster of inner portion of sample had no effect on the impact toughness, probably because the impact load was mainly withstood by the boundary wall.

Table 6 compares the present study with the past studies with respect to the effects of various 3D printing parameters on the impact toughness. The past studies mainly investigated the effect of 3D printing parameters on the impact strength of monolithic sheets, i.e., single sheet printed in one or multiple materials. On the other hand, the present study focused on hybrid composite with laminar structure contrary to the monolithic structure in past studies. Comparing the effect of layer thickness on two types of the structures, the results for monolithic structure made in Santhakumar et al. [25] agrees with that of the laminar structure made in the present study. However, the results reported by Shubham et al. [41] completely contradicts the current study, thereby pointing out the probable role of material type, thereby pointing out the combined effect of parameters and material type on the toughness of 3D-printed structures. With regards to the infill density, its effect is the same in both the monolithic and laminar structures, thereby indicating that nature of the fill density effect remains unchanged regardless of the type of structure or material.

### 3.2. Empirical Model and Optimization

An empirical model was developed to correlate a response with the complete range of conditions of the entire design. For this purpose, the regression analysis was done using DX-10 software. The following empirical model was yielded for the only response, i.e., impact toughness:

Impact toughness = 15,189.29 + 3071.43 × LT + 330.17 × RA + 2343.95 × CR − 1426.83 × FD(7)

Experiments were performed to validate the model, as shown in Table 7. The error in the predicted and experimental values was reported less than 5%, which confirms that the model can likely predict the impact toughness of hybrid C-PLA/ABS laminar composite.

The desirability function as described by Costa [49] was used for optimization:

D = (d_1_^r1^ × d_2_^r2^ …… × d_m_^r.m^)^1/(r1+r2+…+rm)^ (8)

where D = collective desirability of all responses, d_i_ = desirability of individual response, and r_i_ = weightage of each response.

As studied above, the impact toughness of the specimen was affected by the 3D printing parameters. Optimum conditions were developed to attain appropriate impact toughness. Optimization was done with an iterative manner and 100 solutions were achieved. 

For optimization in this study, LT of 0.5 mm, CR of 1, FD of 40%, and RA of 45° were determined to be the best 3D printing parameters. To verify these 3D printing conditions, a sample was 3D printed and tested. The predicted and actual strength were 22,361.7 and 23,465.6 kJ/m^2^, respectively, with a difference of 4.7%. It can be observed that optimized results are in agreement with the experimental results. 

### 3.3. Types of Failure and Fracture Mechanism

**Figure 7:**
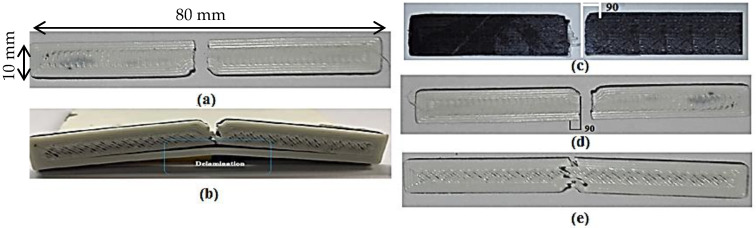
Fractures in C-PLA/ABS hybrid composite: (**a**) Both laminates fractured without any delamination; (**b**) Delamination of boundary wall; (**c**) Brittle fracture in C-PLA lamina with RA of 0°/90° and 45°/−45°; (**d**) Brittle fracture in ABS lamina with RA of 0°/90°; (**e**) Ductile fracture in ABS lamina with RA of 45°/−45°.

As shown in Figure 7a,b, two types of failures were observed in the composite samples, namely, type A and type B. In the type A failure (Figure 7a), both laminates fractured simultaneously as a hammer hit the sample without any delamination. This type of failure was observed in the samples with lower values of CR (0.5) and LT (0.1 mm). In fact, when CR = 0.5, the proportion of brittle C-PLA (67%) was greater than that of ductile ABS (33%). Moreover, when LT was low (say 0.1 mm), the material experiences greater number of thermal cycles thereby leading to interlayer cracking LT = 0.1 mm [1]. These two factors as a result led to brittle failure (i.e., type A). 

The type B failure differed from type A in a sense that it included delamination of ABS boundary wall besides fracturing of remainder composite (Figure 7b). This kind of failure was observed in samples with higher values of CR (1) and LT (0.5 mm). Higher CR means greater volume of ductile ABS than that of brittle C-PLA. Further, high LT reduces the number of thermal cycles, thus reducing interlayer cracking. These conditions as a result promotes nature of fracture from brittle to ductile. This fact is evidenced from Figure 7b, whereby the failure is dictated by delamination and zigzag fracture. Out of 16 samples produced in this work, 12 samples failed according to the type A and 4 failed according to the type B as listed in Table 8.

Further, it was observed that the C-PLA lamina of the composite always fractured in a brittle manner making an angle of 90° with the loading axis for both of 0°/90° and 45°/−45° rasters (Figure 7c). The fracture in ABS lamina, however, showed dependence on the raster angle: fractured at an angle of 90° for the raster of 0°/90° and at about 40° (with respect to loading axis) for the raster of 45°/−45° as shown in Figure 7d,e. In other words, as observable from Figure 7d,e, the fracture in ABS lamina tends to propagate along the raster direction.

**Figure 8:**
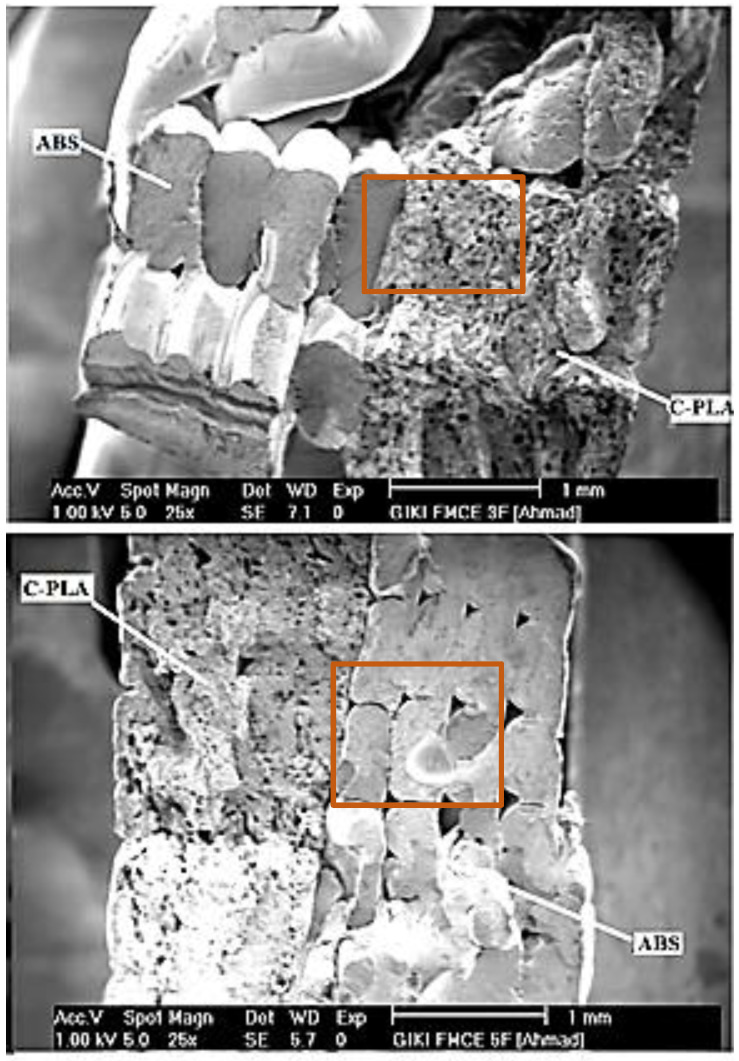
SEM images of cross sections of fractured sample 3 and sample 5.

Figure 8 shows the SEM images of fractured surfaces of the representative samples of C-PLA/ABS laminar composite (i.e., 3 and 5). In the case of fractured C-PLA sheet, two types of voids/pores can be noticed. Large voids, which have very low density, represent absence of material flow during printing. Small voids, which have much higher density, signify breaking and pull out of carbon fibers, thereby suggesting that failure in the C-PLA sheet initiated probably due to breaking of fibers once the impact load exceeded the fiber strength (Figure 8). The breaking of both fiber and PLA matrix indicates that the impact load was jointly borne by the two with main contributions from the fibers. Further, no interlayer cracking is observed in the C-PLA sheet showing that the printed layers remained intact during impact loading because of strong interlayer adhesion. On the other hand, interlayer cracking can be observed in the ABS lamina, specifically near the pores (Figure 8), thereby indicating poor interlayer adhesion and suggesting that breaking of the ABS beads might had initiated from the pores. Such cracks can be also observed at the C-PLA/ABS interface. In case of ABS lamina, the entire load was borne by the ABS matrix contrary to matrix and fibers in the C-PLA sheet.

## 4. Conclusions

In this study, ABS polymer was laid upon the 3D-printed C-PLA lamina using FFF technology with an objective to improve its impact toughness. This printing configuration led to the fabrication of a hybrid laminar composite. The composite was produced by varying FFF conditions. Impact tests were performed to find out the impact toughness of the fabricated samples. Several important conclusions were drawn from this study as outlined below:

1. Boundary wall is found to increase the impact toughness of FFF-built samples. An increase of 60% was obtained due to making boundary wall around the sample.
2. The strategy of coupling ABS lamina with the C-PLA lamina substantially increases the impact toughness (280 to 365%) as compared to that of the equivalent C-PLA lamina.
3. A change in the 3D printing parameters affects the impact toughness of the hybrid laminar composite, except raster angle appears to be insignificant. The impact toughness is found to range from 7672.9 to 23,465.6 kJ/m^2^ in this study. The optimum conditions sought for maximizing the impact toughness suggest that greater layer thickness (0.5 mm), lower fill density (40%), and higher clad ratio (1) are conducive.
4. Two different types of failures are observed when the hybrid laminar composite is subjected to impact load. In the type A failure, both laminates fractured simultaneously without any delamination as hammer hit the sample. The type B failure differed from type A in a sense that it included delamination of ABS boundary wall besides fracturing of the remainder composite.
5. Voids in the fractured surface of C-PLA lamina were observed due to absence of material and breaking of fibers, while voids in the ABS fractured surface were noticed only due to absence of material. Further, interlayer cracks were observed in the ABS fractured surface near the pores suggesting poor interlayer adhesion. While, no such crack was observed in the C-PLA fractured surface, indicating higher interlayer adhesion.

## Figures and Tables

**Table 1:** Properties of ABS and C-PLA filaments.

Property	ABS	C-PLA
Tensile modulus (MPa)	2241	6560
Flexural strength (MPa)	43	96
Melting point (°C)	N/A (amorphous)	150–180 (for 4043D PLA)
Glass transition temperature	105	60

**Table 2:** Impact toughness of samples made with and without boundary wall.

Materials	Impact Toughness without Boundary Wall	Impact Toughness with Boundary Wall	Improvement
-	kJ/m^2^	kJ/m^2^	%
ABS	18,963	25,035	32
Composite	14,596	23,466	60
C-PLA	3855	5044	30

**Table 3:** Experimental plan and Impact Toughness Results of hybrid laminar composite.

Test No.	LT	RA	CR	FD	Impact Toughness
-	mm	Degree(°)	-	%	kJ/m^2^
1	0.5	0/90	1	40	22,874.35
2	0.1	0/90	1	40	17,468.75
3	0.5	45/−45	1	40	23,465.62 (max)
4	0.1	0/90	1	100	13,162.43
5	0.5	45/−45	1	100	16,017
6	0.5	0/90	0.5	100	11,762.18
7	0.1	45/−45	0.5	100	9017.5
8	0.5	0/90	0.5	40	16,016.87
9	0.1	0/90	0.5	40	10,380.78
10	0.5	45/−45	0.5	40	17,471.39
11	0.5	0/90	1	100	18,943.43
12	0.1	45/−45	1	40	13,162.43
13	0.1	45/−45	1	100	14,580.62
14	0.1	0/90	0.5	100	7672.87 (min)
15	0.1	45/−45	0.5	40	11,497.53
16	0.5	45/−45	0.5	100	18,943.62

**Table 4:** Improvement by laying ABS on C-PLA sheet.

Test No.	C-PLA	Hybrid Composite	Improvement
	kJ/m^2^	kJ/m^2^	%
3 (max)	5044	23,465	365
14 (min)	2019	7672	280

**Table 5:** ANOVA for reduced 2FI model of Impact Toughness.

Source	Sum of Square	Df	Mean Square	F Value	*p*-Value Prob > F	Significant
Model	2.822 × 10^8^	5	5.644 × 10^7^	15.27	0.0002	√
A-LT	1.473 × 10^8^	1	1.473 × 10^8^	39.85	<0.0001	√
B-Ang	2.157 × 10^6^	1	2.157 × 10^6^	0.5833	0.4627	×
C-CR	8.516 × 10^7^	1	8.516 × 10^7^	23.03	0.0007	√
D-FD	3.091 × 10^7^	1	3.091 × 10^7^	8.36	0.0161	√
BC	1.665 × 10^7^	1	1.665 × 10^7^	4.50	0.0598	×
Residual	3.697 × 10^7^	10	3.697 × 10^6^			
Core Total	3.192 × 10^8^	15				

**Table 6:** Comparison between the present and past studies.

Study	3D Printed Structure	LT	FD	CR
Current	ABS/C-PLA hybrid laminar composite	Impact toughness increases with LT from 0.1 to 0.5 mm	Impact toughness increases with decreasing FD from 100 to 40%	Impact toughness increases with increasing CR from 0.5 to 1
Shubham et al. [41]	ABS monolithic sheet	Impact strength decreases (54%) with increasing LT from 0.075 to 0.5 mm	--	--
Santhakumar et al. [44]	Polycarbonate monolithic sheet	Impact strength increases with LT increasing from 0.18 to 0.25 mm	--	--
Joseph et al. [45]	Thermoplastics monolithic sheets	--	Impact strength increases with decreasing FD up to 25%	--
Isfahani et al. [46]	Polyester fiber monolithic composite sheet	--	Impact strength increases with decreasing FD up to 20%	--

**Table 7:** Experimental vs. predicted value.

LT	RA	CR	FD	Actual Strength	Predicted Strength	% Error	Failure Type
mm	Degree	-	%	kJ/m^2^	kJ/m^2^	-	-
0.1	45/−45	1	40	13,162.5	13,759.5	−4.3	Type B
0.5	45/−45	1	66	21,982.3	21,112.4	+4.1	Type B
0.5	0/90	0.9	40	19,989.3	20,676	−3.32	Type A

Note: Type A and Type B defined in Section 3.3.

**Table 8:** Facture type and nature of samples.

Sample No.	Failure Type	Fracture Nature	
		ABS	C-PLA
1	B	Brittle	Brittle
2	A	Ductile	Brittle
3	B	Ductile	Brittle
4	A	Brittle	Brittle
5	B	Brittle	Brittle
6	A	Brittle	Brittle
7	A	Brittle	Brittle
8	A	Brittle	Brittle
9	A	Ductile	Brittle
10	A	Ductile	Brittle
11	B	Brittle	Brittle
12	A	Ductile	Brittle
13	A	Ductile	Brittle
14	A	Brittle	Brittle
15	A	Ductile	Brittle
16	A	Ductile	Brittle

Note: Type A and Type B defined in Section 3.3.
